# Challenges and complexities of multifrequency atomic force microscopy in liquid environments

**DOI:** 10.3762/bjnano.5.33

**Published:** 2014-03-14

**Authors:** Santiago D Solares

**Affiliations:** 1 Department of Mechanical Engineering, University of Maryland, College Park, MD 20742, USA

**Keywords:** amplitude-modulation, bimodal, frequency-modulation, liquids, multifrequency atomic force microscopy

## Abstract

This paper illustrates through numerical simulation the complexities encountered in high-damping AFM imaging, as in liquid enviroments, within the specific context of multifrequency atomic force microscopy (AFM). The focus is primarily on (i) the amplitude and phase relaxation of driven higher eigenmodes between successive tip–sample impacts, (ii) the momentary excitation of non-driven higher eigenmodes and (iii) base excitation artifacts. The results and discussion are mostly applicable to the cases where higher eigenmodes are driven in open loop and frequency modulation within bimodal schemes, but some concepts are also applicable to other types of multifrequency operations and to single-eigenmode amplitude and frequency modulation methods.

## Introduction

Multifrequency atomic force microscopy (AFM) refers to a family of techniques that involve simultaneous excitation of the microcantilever probe at more than one frequency [1]. The first of these methods was proposed by García and coworkers in 2004 to carry out simultaneous non-contact amplitude-modulation imaging and open-loop (phase contrast) compositional mapping of surfaces in air by exciting and controlling the first two eigenmodes of the cantilever [2]. This approach has since been extended to intermittent contact characterization using open loop and frequency modulation [3–4], imaging in liquid and vacuum environments [5–8], and to trimodal operation [9–11]. There also exist a number of other multifrequency and multiharmonic AFM techniques which have been developed for different purposes [1,12–18].

**Figure 1:**
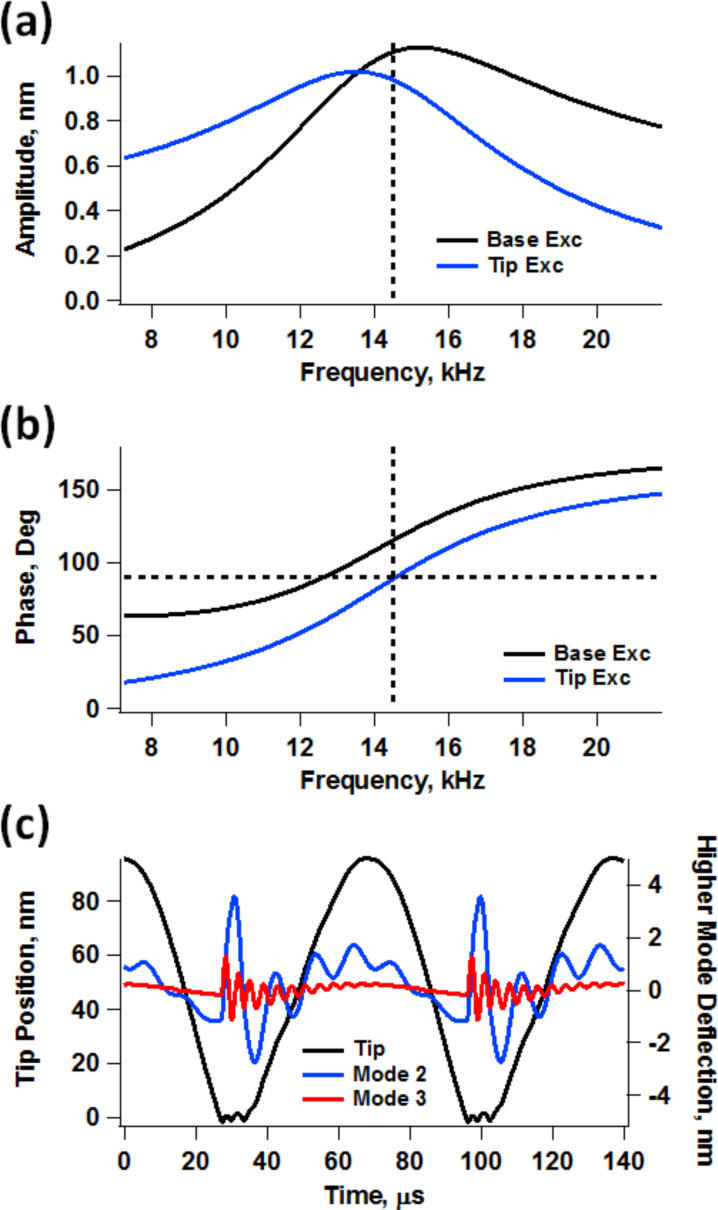
Example of measurement artifacts previously observed in single-mode AFM operation in liquids: distortion of the frequency response (a) and phase response (b) curves with base excitation (the “Tip Exc” traces provide the true response); momentary excitation of higher eigenmodes and multiple tip–sample impacts for every cycle of the fundamental eigenmode (c). The simulation parameters are ν_1_ = 14.5 kHz, *k*_1_ = 0.03 N/m, *Q*_1_ = 2, *Q*_2_ = 6, *A*_free_ = 75 nm, *A*_setpoint_ = 55% and sample modulus of elasticity of 2 GPa (Hertzian contact).

Previous researchers have shown that the dynamics of the AFM cantilever become extremely complex for low-*Q* environments, such as liquids [19–28] (see Figure 1), and have identified phenomena such as the momentary excitation of higher eigenmodes and multiple-impact regimes [21,26], mass loading and fluid-borne cantilever excitation [19,23–24], discrepancies between the photodetector signal and the actual tip position for base-excited cantilever systems [24,28] and non-ideal spectroscopy curves (for example, curved amplitude–distance curves where multiple regimes are observed as kinks [19]). Although the focus of these studies has not been on techniques designed for driving the cantilever at different frequencies simultaneously, it is not surprising that all of the above phenomena are also present in multifrequency operations and that the various issues compound with the added complexity of multifrequency AFM [9,29–32], such that more and more experience and knowledge is required from the user to carry out meaningful measurements. With multifrequency methods it can be more difficult to achieve suitable imaging conditions and to properly interpret the results, and no single recipe works in all cases. This paper explores through simulation the implications of the low-*Q* cantilever dynamics within the specific context of bimodal AFM imaging. The primary focus is on (i) the amplitude and phase “relaxation” (equilibration) for driven higher eigenmodes between successive taps of the fundamental eigenmode regardless of the point of application of the excitation (base or tip), (ii) momentary excitation of non-driven eigenmodes, and (iii) additional artifacts introduced by the use of base excitation. The discussion is most directly applicable to bimodal techniques where the higher eigenmode is driven in open loop [5,8] or frequency modulation [4], but the principles are general enough that they are also relevant to other multifrequency methods and in some cases also to single-mode frequency and amplitude modulation techniques. Finally, it is noted that some of the challenges discussed here, namely those caused by sharp variations in the tip–sample forces can be mitigated through the use of small-amplitude operation [7–8], although this may not always be feasible, depending on the type of sample and the type of instrument that is available.

## Results and Discussion

### Amplitude and phase relaxation of driven eigenmodes

**Figure 2:**
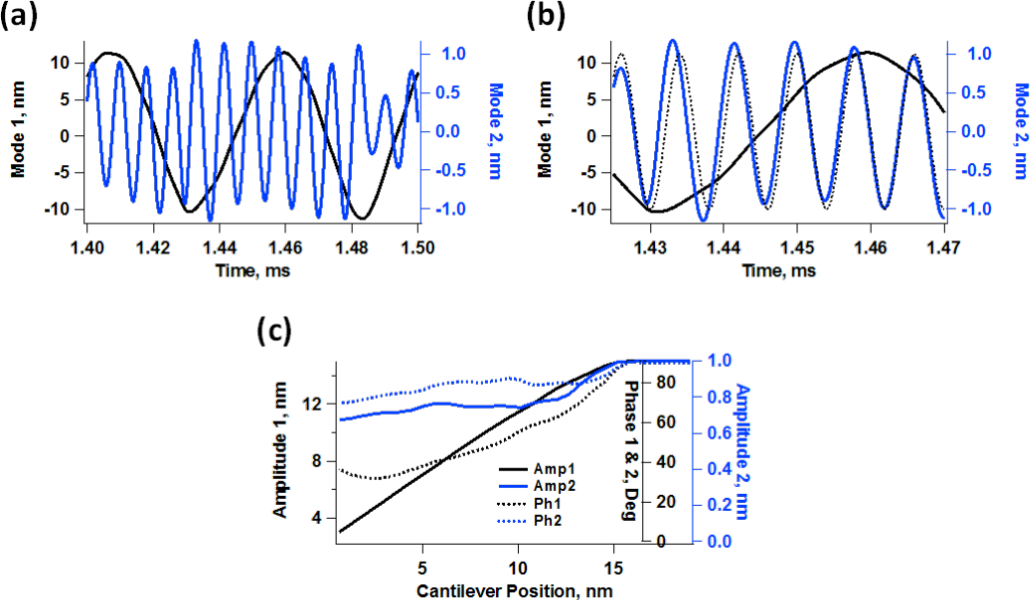
Bimodal AFM simulation illustrating the phase and amplitude relaxation of the second eigenmode: (a) different response of the first and second eigenmode over successive tip–sample impacts (successive tip–sample impacts are different because the ν_2_/ν_1_ ratio is generally not an integer); (b) phase relaxation of the second eigenmode (the dotted line shows the fully relaxed response – notice how this eigenmode’s response gets ahead with respect to the dotted line and undergoes a change in amplitude after the impact but then recovers before the next tap); (c) seemingly normal amplitude and phase spectroscopy curves. The cantilever parameters are ν_1_ = 20 kHz, *k*_1_ = 0.25 N/m, *Q*_1_ = 3, *Q*_2_ = 6, *A*_free_ = 15 nm, and *A*_setpoint_ = 70% (a and b only). The sample was modeled as a standard linear solid (see methods section) with *K*_o_ = 3.5 N/m, *K*_inf_ = 3.5 N/m and *C*_d_ = 1 × 10^−5^ Ns/m.

Previous work by Raman and coworkers [22] demonstrated that in high-damping environments the phase contrast derives primarily from an “energy flow channel” that opens up when higher modes of the cantilever are momentarily excited through the tip–sample impact (see Figure 1c), which is more prevalent for softer cantilevers than for harder ones. When this happens, the phase contrast does not map dissipation, but instead short-range conservative interaction variations. The phenomenon is called momentary excitation because the oscillation of the higher eigenmodes begins with the tip–sample impact, governed by the frequency and amplitude of the fundamental eigenmode, and decays in between successive taps of the cantilever. This fast decay occurs because the quality factor of the higher eigenmodes is generally smaller than the ratio of eigenfrequencies [21,26]. In the case of bimodal AFM, a similar phenonmenon takes place, where the driven higher eigenmode is perturbed every tip–sample impact and the perturbation relaxes in between successive taps. However, the situation is slightly different since the eigenmode is also being actively driven with a sinusoidal excitation. Here the perturbation appears to the user as a momentary variation in the phase and amplitude of the higher mode (see Figure 2a and 2b), which relaxes until the phase and amplitude reach the values they would have in the absence of the sample, just before the next impact occurs. This rich behavior is not captured in the phase and amplitude signals (see Figure 2c), which are obtained through averaging over multiple oscillation cycles. However, such behavior can preclude the application of the phase spectroscopy theories that have been developed for operation in air environments, which assume a nearly-equilibrated eigenmode oscillation where all cycles are sinusoidal and similar in phase and amplitude [33–34].

**Figure 3:**
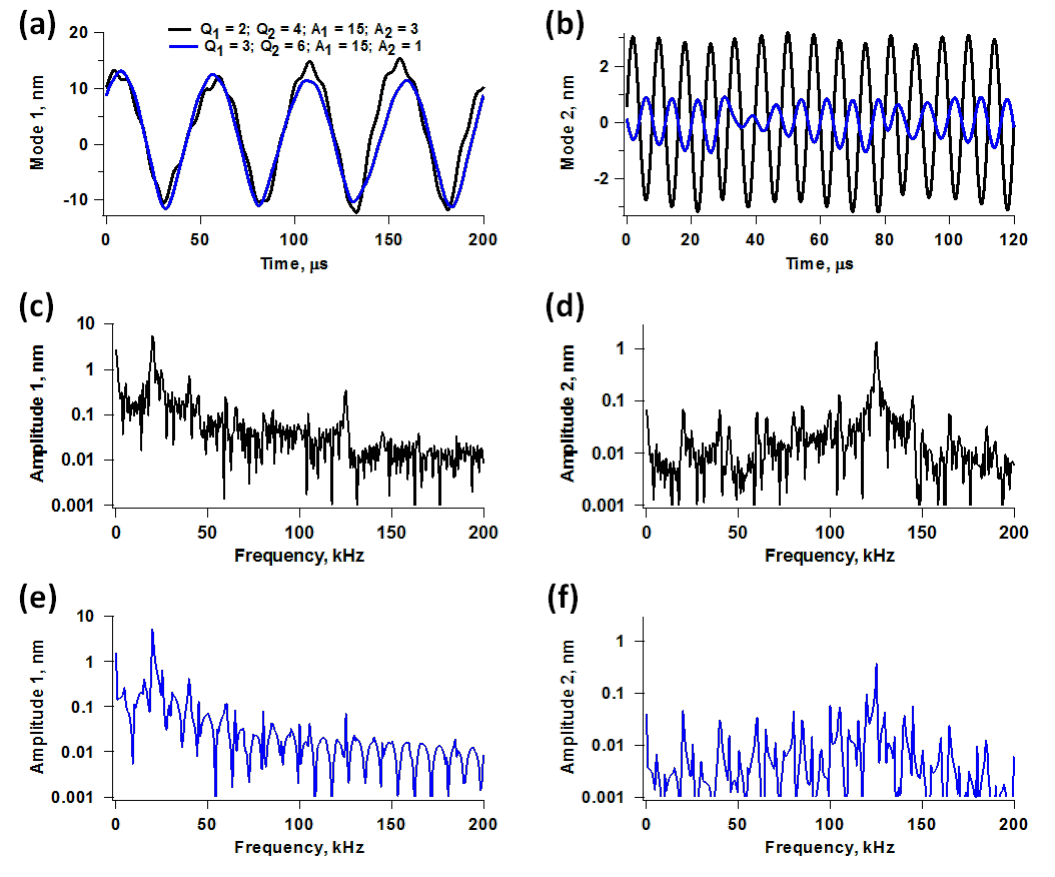
Illustration of eigenmode perturbation for two different cases. The results are color coded for the two cases considered, *Q*_1_ = 2, *Q*_2_ = 4, *A*_1_ = 15 nm and *A*_2_ = 3 nm for case 1 (black traces), and *Q*_1_ = 3, *Q*_2_ = 6, *A*_1_ = 15 nm and *A*_2_ = 1 nm for case 2 (blue traces). (a) mode 1 responses in time space; (b) mode 2 responses in time space; (c) and (d) mode 1 and mode 2 spectra, respectively, for case 1; (e) and (f) mode 1 and mode 2 spectra, respectively, for case 2. The amplitude setpoint is approximately 70% and the sample parameters are the same as for Figure 2. Notice how the use of a smaller value of *A*_2_ results in a sharper spectrum for the first mode but a less sharp spectrum for the second mode, and vice-versa.

**Figure 4:**
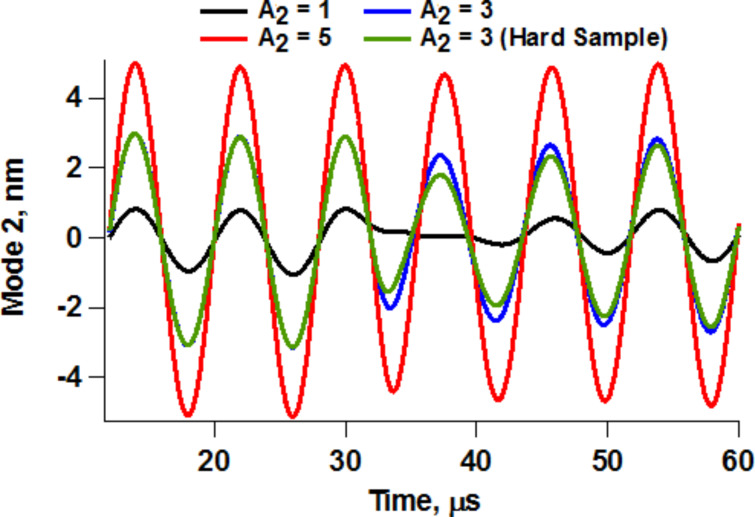
Second eigenmode response for different second mode free amplitude values for the same conditions as the simulations in Figure 3, and for a stiffer sample with *K*_o_ = 7 N/m and *K*_inf_ = 7 N/m (green trace), which causes greater perturbation for a given amplitude.

**Figure 5:**
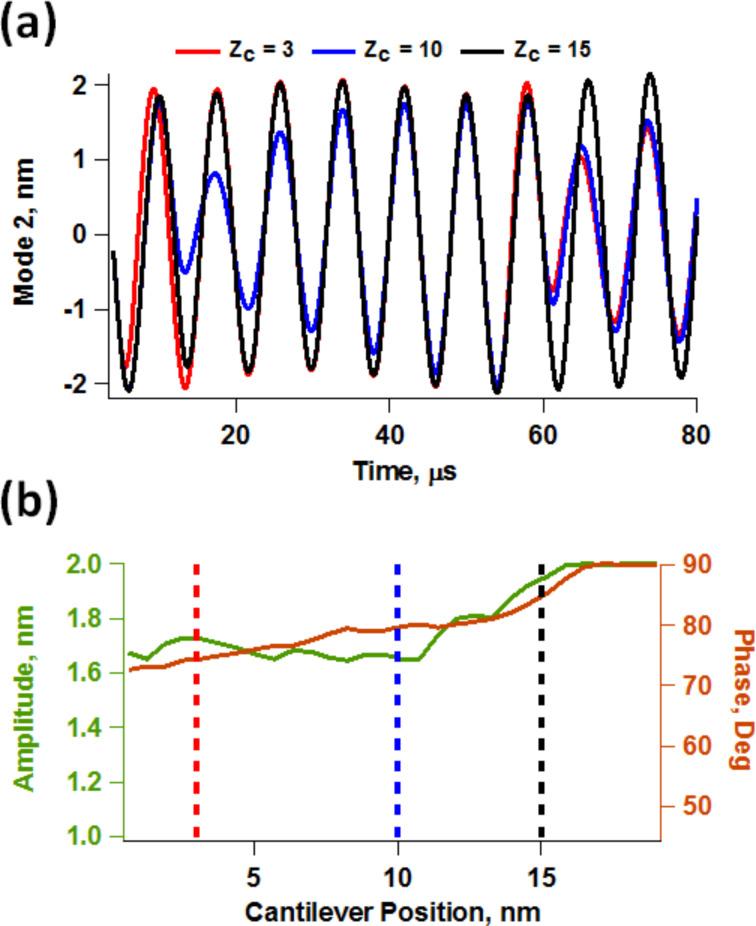
(a) Illustration of the drastically varying response of the higher eigenmode as the cantilever is brought closer to the sample (*Z*_c_ denotes the cantilever position above the sample); (b) second phase response for the three responses shown in (a).

Due to the short equilibration times in liquids, in bimodal operation the response of the cantilever eigenmodes exhibits a distinct transient and a relaxed contribution. The relaxed contribution is equal to the eigenmode’s response in the absence of the sample. The transient contribution is a result of the forces that take place during each impact. The ability of these forces to modify the response of each individual eigenmode is strongly dependent on the imaging conditions. This is illustrated in Figure 3 for two cases involving different quality factor and higher mode amplitudes. In general, higher modes are more likely to be perturbed when their free amplitude is small (discussions on this topic can be found in references [8,11]). However, the oscillation of the fundamental eigenmode is more likely to be perturbed with larger amplitudes of the higher eigenmode due to a more irregular impact. This is also illustrated in Figure 3, which includes real-time trajectories and frequency space representations of the first two eigenmode responses. The two cases analyzed correspond to slightly different values of the quality factors, but their effect was not significant in the range considered. Figure 4 shows a more direct comparison of the second eigenmode response under similar conditions for different free amplitudes, providing also an example for a ‘harder’ sample. As it is well known, stiffer samples are more likely to perturb the oscillation of a given cantilever. This is extremely important, as samples with inhomogeneous stiffness can give rise to different types of perturbations across the surface, such that quantitative interpretations of the contrast across the entire sample may become meaningless. Furthermore, the level of cantilever perturbation is also highly sensitive to the amplitude setpoint, as illustrated in Figure 5 for three different cantilever positions above the sample. Clearly, the oscillation changes significantly as the cantilever is lowered towards the sample (Figure 5a), even though the average phase and amplitude response do not exhibit drastic variations (Figure 5b). This is highly relevant when carrying out quantitative comparisons for different types of samples, which may require individual optimization of the imaging conditions.

**Figure 6:**
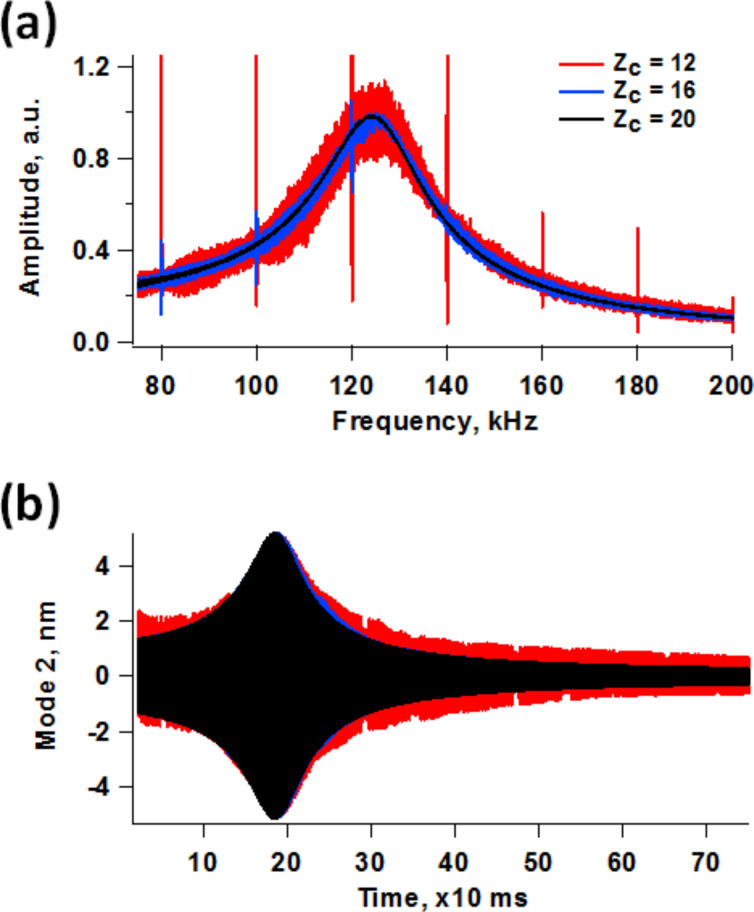
Frequency space (a) and time space (b) responses of the system of Figure 5 for three different cantilever positions above the sample, obtained by sweeping the frequency from low to high using a chirp function while keeping the cantilever at the fixed height indicated. *Z*_c_ = 20 nm corresponds to the free response. The results shown in (a) were obtained through application of the fast Fourier transform to the results shown in (b).

**Figure 7:**
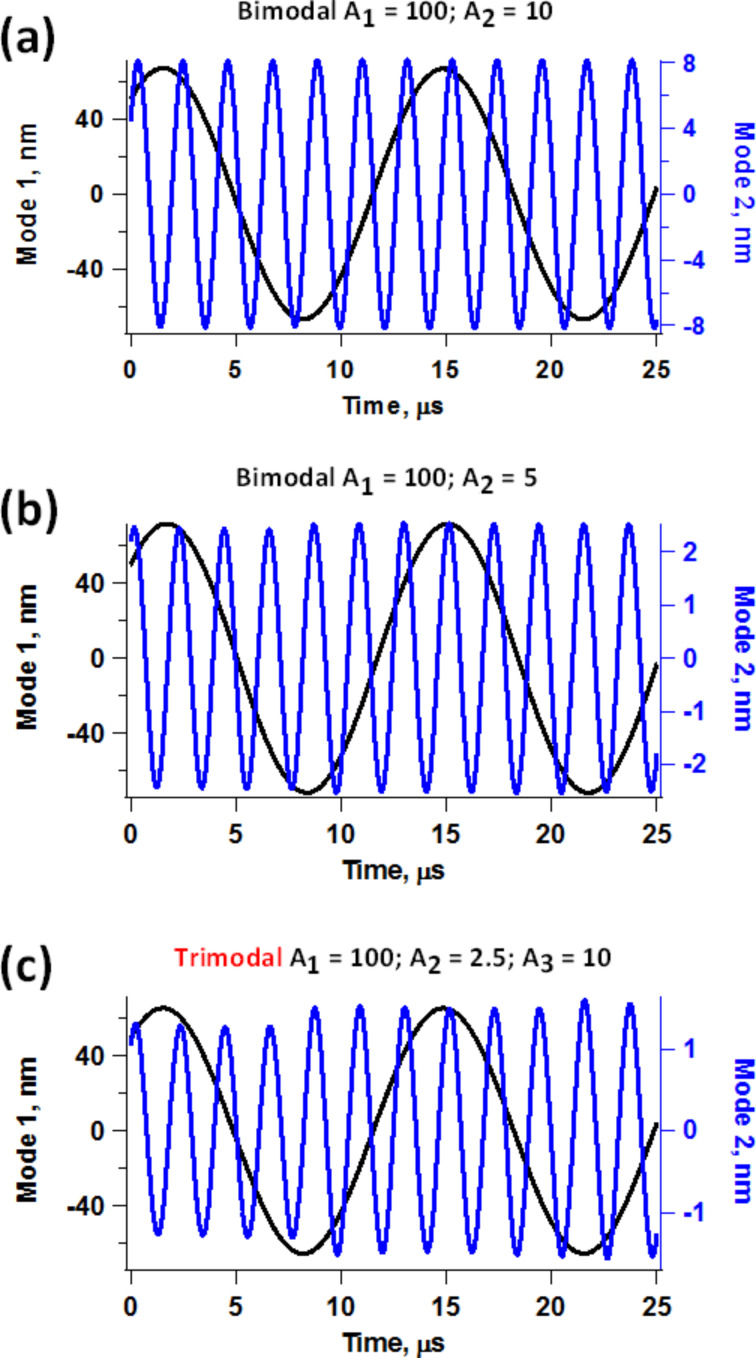
Typical eigenmode responses for bimodal and trimodal AFM operation in air with *Q*_1_ = 150, *Q*_2_ = 450, *Q*_3_ = 750, ν_1_* =* 70 kHz, ν_2_* =* 437.5 kHz, ν_3_* =* 1.25 MHz and *k*_1_ = 2 N/m: (a) bimodal operation with *A*_1_ = 100 nm and *A*_2_ = 10 nm (*A*_1_/*A*_2_ = 10); (b) bimodal operation with *A*_1_ = 100 nm and *A*_2_ = 5 nm (*A*_1_/*A*_2_ = 20); (c) trimodal operation with *A*_1_ = 100 nm and *A*_2_ = 2.5 nm (*A*_1_/*A*_2_ = 40 and *A*_3_/*A*_2_ = 4). The sample parameters were *K*_o_ = 10 N/m, *K*_inf_ = 10 N/m and *C*_d_ = 1 × 10^−5^ Ns/m.

The phenomena introduced by the higher eigenmode phase and amplitude relaxation within an oscillation cycle of the fundamental eigenmode bring about obvious challenges in the interpretation of phase contrast images. However, the difficulties become even more significant if one wishes to implement bimodal operations involving frequency modulation (FM) control of the higher eigenmode [4]. While the phase contrast results may become less and less meaningful as momentary perturbations become more and more severe, one is still generally able to obtain an image with open loop drive of the higher mode. However, the implementation of FM requires either a phase-locked loop (PLL) or time delay (phase shifting), both of which are more complex and highly sensitive to perturbations. The time delay version of FM is even more susceptible to instabilities because the excitation of the cantilever is created from the real-time response of the cantilever, one cycle at a time. Figure 6 shows frequency and time domain second eigenmode responses obtained by sweeping the excitation frequency from low to high using chirp functions [35] while keeping the cantilever at a fixed height above the sample within bimodal operation. The trace for a cantilever height *Z*_c_ = 20 nm is the free (unperturbed) response away from the sample. As the cantilever is lowered (*Z*_c_ = 16 nm and *Z*_c_ = 12 nm), the response becomes noisier, although it still retains its general Lorentzian behavior, suggesting that FM control may still be possible if sufficient signal averaging is performed. While the time delay version of FM may be impractical due to the cycle-to-cycle variations in the phase and amplitude, PLL operation may still be feasible, since the latter is based on the calculation of the average instantaneous phase which the system attempts to gradually lock to a specific value according to user-defined gains. However, even in this case the results may or may not be meaningful and characterization may be undesirably slow, depending on the severity of the perturbations induced by the tip–sample forces. The situation becomes more favorable as the higher mode quality factor increases such that the phase and amplitude relaxation becomes slower and intermixing of transients from different cycles occurs, similar to what happens in air environments. Specifically, for the *i*-th higher eigenmode it would be necessary that its quality factor be significantly greater than the ratio ν*_i_*/ν_1_ such that the transients extend appreciably beyond one cycle of the fundamental frequency (here ν_1_ is the fundamental eigenfrequency and ν*_i_* is the higher mode eigenfrequency). For some applications, there may exist cantilevers that meet these requirements and in other cases it may be possible to utilize high-*Q* techniques designed for characterization in liquids, such as the recently proposed trolling mode method [36]. For comparison purposes Figure 7 shows typical second eigenmode responses for bimodal and trimodal operation in air. Even for the trimodal case, which corresponds to a very drastic situation in which the second eigenmode amplitude is very small compared to the fundamental amplitude and four times smaller than the third mode amplitude, the response is much more regular than for the results discussed above for liquid imaging.

### Momentary excitation of non-driven eigenmodes

**Figure 8:**
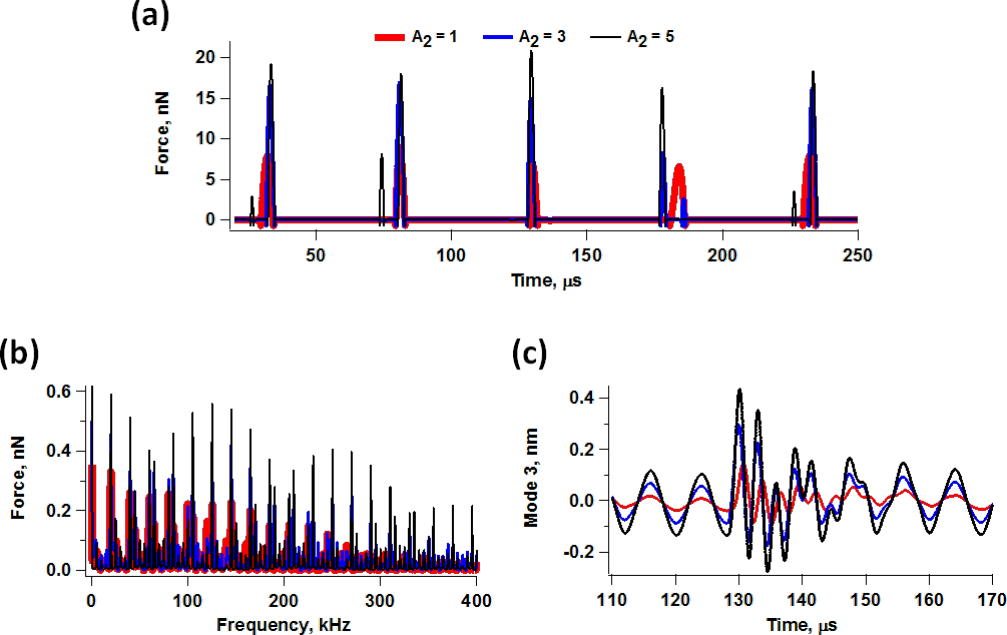
Illustration of the force trajectory of five successive tip–sample impacts for bimodal AFM conditions similar to those used to construct Figure 2, for three different second mode amplitudes ranging from 1 nm to 5 nm (a) along with the corresponding force spectra (b) and typical third eigenmode momentary excitation responses (c).

While the previous section focuses on the momentary perturbation of the driven higher eigenmodes, one must still be mindful of the momentary excitation of non-driven eigenmodes, since both phenomena have the same underlying cause. As extensively studied through simulation and experiment by Raman and coworkers, momentary excitation occurs when the spectrum of the tip–sample forces overlaps with the frequency response (transfer) function of the higher eigenmodes, which is more likely to occur in low-*Q* environments for which the eigenmode bandwidth is greater [21,26]. This phenomenon also occurs in multifrequency AFM with the added complexity that the tip–sample forces depend strongly on the parameters chosen to drive the higher eigenmodes, as well as on their nonlinear interaction with the fundamental eigenmode oscillation. As a result, the observed momentary excitation of non-driven eigenmodes will also depend strongly on the driven higher eigenmode parameters. Figure 8a shows five successive force trajectories for bimodal operation using similar conditions and for a similar sample as for Figure 2, for three different second mode amplitudes. As expected, there is a significant change in tip–sample penetration as the second mode amplitude increases [11,37], leading to different force spectra (Figure 8b). Since all three spectra overlap at least with the third eigenmode frequency response, they all lead to its momentary excitation to different degrees, as shown in Figure 8c. Furthermore, in contrast to single-mode operation, the momentary excitation can differ significantly for successive fundamental eigenmode oscillations (not shown). This is because the ratio of the second to the first eigenfrequency is not an integer, which leads to different successive impacts. Since the third eigenmode is an “energy channel” separate from the two driven eigenmodes [22], its momentary excitation leads to changes in the response of the other two modes in a manner which is not easily predictable a priori. Some generalization is possible, but since there is no single interpretation that applies in all cases, monitoring of the higher mode responses, as well as user experience and discretion are critical for studies that go beyond simple qualitative observations.

### Base excitation and cantilever tuning artifacts

**Figure 9:**
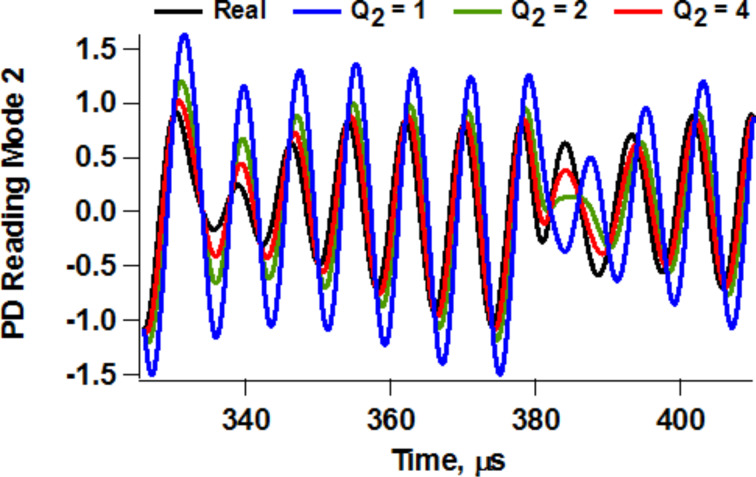
Illustration of the photodetector (PD) reading that would be obtained for a given second eigenmode trajectory (taken from a tip-excited bimodal simulation) for different values of the quality factor when the cantilever is driven using base excitation. There is a clear discrepancy between the photodetector reading and the real trajectory as *Q* drops.

The differences between base- and tip-excited systems have also been previously discussed for single-mode operation [19,24,28], but as for the issues discussed in the two previous sections, they are worth revisiting here in the specific context of multifrequency AFM. These differences are not extremely relevant for simple imaging applications, but they are critical when a higher eigenmode is used to carry out compositional mapping while imaging. While most of the AFM systems in use only have base excitation capability, it is important to keep in mind the fact that unless the cantilever base motion is known with high accuracy (unfortunately this is not practical and only possible within highly controlled experiments) and the cantilever behaves in an ideal manner, it is not possible to determine the true tip trajectory from the photodetector reading. This is because the photodetector measures cantilever deflection (this can be approximated as tip position minus base position), not tip position. Figure 9 illustrates the photodetector readings that would be obtained for different values of the quality factor for a given second eigenmode tip oscillation (labeled as “Real”). Clearly spectroscopic measurements are not meaningful unless the true probe trajectory is known. This is a challenge that remains unsolved even in the most sophisticated base-excitation experiments, which is further compounded by the non-ideal behavior of piezo shaker systems, cantilevers and the surrounding fluid [19,23–24]. One obvious consequence of this difficulty is that tip–sample dissipative and conservative forces cannot be measured accurately with base-excited systems when the effective quality factor of the cantilever changes throughout the measurement. In such cases, the phase of the oscillation would artificially change as tip–sample dissipation changes, leading to inaccurate readings. In frequency modulation operation this would cause the system to lock to a varying (non-constant) phase, which would render the results meaningless. Accurate measurements of this type with base-excited systems would only be possible if one carries out volume scanning above the surface, running a full frequency sweep curve at each grid point and fitting it to the appropriate base excitation response curve [28]. This practice is not the norm and would be time consuming, but is not necessarily out of reach since the transient times in liquid are short and the measurements can be carried out much more rapidly than in air or vacuum.

**Figure 10:**
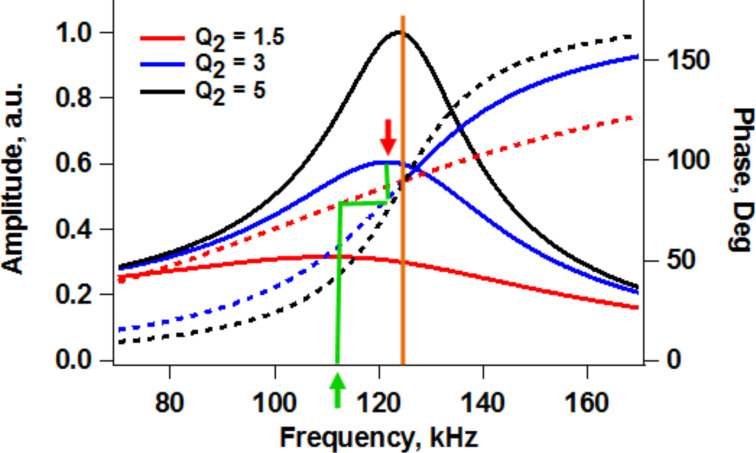
Cantilever amplitude and phase response for various levels of damping in low-*Q* environments. The thick red and green arrows and the green line illustrate the nature of the errors made in determining the resonance frequency when a frequency modulation operation is locked to the peak frequency instead of the natural frequency (see discussion in the text).

In the cases where frequency modulation operation can still be stably implemented with tip excitation whether for single- or multimode operation, it is important to note that the phase of the oscillation must be locked to 90 degrees during tuning even if this does not correspond to the amplitude peak (this is true for tip-driven systems and compounds itself with the previously discussed complexities of base excitation). This is because the peak frequency in low-*Q* environments shifts significantly to lower frequencies (see Figure 10), while the frequency at which the phase is 90 degrees remains at the natural frequency. The natural frequency is the only frequency at which all the phase curves intersect for a given (ideal) cantilever driven in environments with different levels of damping (see Figure 10). The type of errors introduced when locking the phase to that of the peak frequency can also be understood using Figure 10. Consider the case when the phase is locked to the maximum response amplitude for a cantilever driven in an environment such that *Q* = 3 (blue traces). The frequency of the peak is indicated by a thick red arrow on the graph (notice that this frequency is to the left of the natural frequency), and the corresponding phase can be found by following the vertical green line downwards until it intersects the phase response for this value of the quality factor. Now, if the level of tip–sample dissipation changes due to tip–sample interactions, such that the effective quality factor drops to 1.5, the phase will remain locked at the same value, but now the phase response of the system will follow a different curve (red dotted line). If one now follows the horizontal green line towards the left until it intersects the new phase response and then draws a vertical line downwards to find the corresponding frequency (thick green arrow), it is clear that the eigenmode will now be driven at a different frequency, leading to the incorrect conclusion that there has been a change in the nature of the conservative forces (since only the dissipative forces have changed). The user will conclude that there has been a frequency shift, when this is clearly not the case. These issues also occur in amplitude modulation AFM and can lead to phase shift measurements that are not quantitatively meaningful.

One final issue to consider for base excited AFM systems is the well-known “forest of peaks” observed during tuning of the cantilever, which makes the selection of the imaging eigenmode difficult. This is even more problematic in multifrequency operation, where one needs to select more than one eigenmode and where the ratio of their eigenfrequencies has important implications with regards to sensitivity. Furthermore, the observed peaks do not generally exhibit a “clean” Lorentzian response, which can render the assumption of harmonic oscillator dynamics questionable. Finally, this non-Lorentzian behavior may also complicate the calibration of the photodetector sensitivity (in V/nm), since there is no guarantee that the selected eigenmodes have the assumed shape. As with various other issues discussed in this document, there is no single answer that fits all situations. Instead, the operator must rely on careful observation and experience in assessing the appropriateness of the eigenmode selection, and must also carefully calibrate the system.

## Conclusion

The key non-idealities observed in low-*Q* AFM have been discussed in the context of multifrequency operation, where additional complexities emerge due to the interaction of the driven and non-driven eigenmodes with one another. A number of challenges have been identified, which are mostly related to open loop and frequency modulation control of the higher eigenmode, and which users should be mindful of when carrying out characterization, especially in the cases where quantitative interpretation of the results is desired. While the focus has been on identifying nonidealities without providing simple or complete solutions, the objective is not to paint a bleak picture of the technique, but rather to raise awareness of open research questions that require further attention within multifrequency AFM.

### Methods

For the numerical simulations three eigenmodes of the AFM cantilever were modeled using individual equations of motion for each, coupled through the tip–sample interaction forces as in previous studies [9,38]. Driven eigenmodes were excited through a sinusoidal tip force or base displacement of constant amplitude and frequency equal to the natural frequency. Chirp excitation functions [35,39] were used to construct the amplitude vs frequency curves, where applicable. Most of the simulations for liquid environment used quality factor values in the range *Q*_1_ = 1–7, *Q*_2_ = 2*Q*_1_–3*Q*_1_; *Q*_3_ = 3*Q*_1_–5*Q*_1_. The equations of motion were integrated numerically and the amplitude and phase of each eigenmode were calculated using the customary in-phase (*I*) and quadrature (*Q*) terms:

[1]
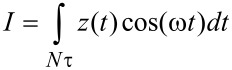


[2]
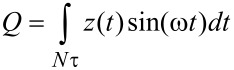


where *z*(*t*) is the eigenmode response in the time domain, *N* is the number of periods over which the phase and amplitude were averaged, ω is the excitation frequency, and τ is the nominal period of one oscillation. The amplitude and phase can be calculated, respectively, as:

[3]
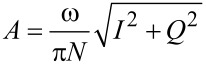


[4]
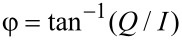


**Figure 11:**
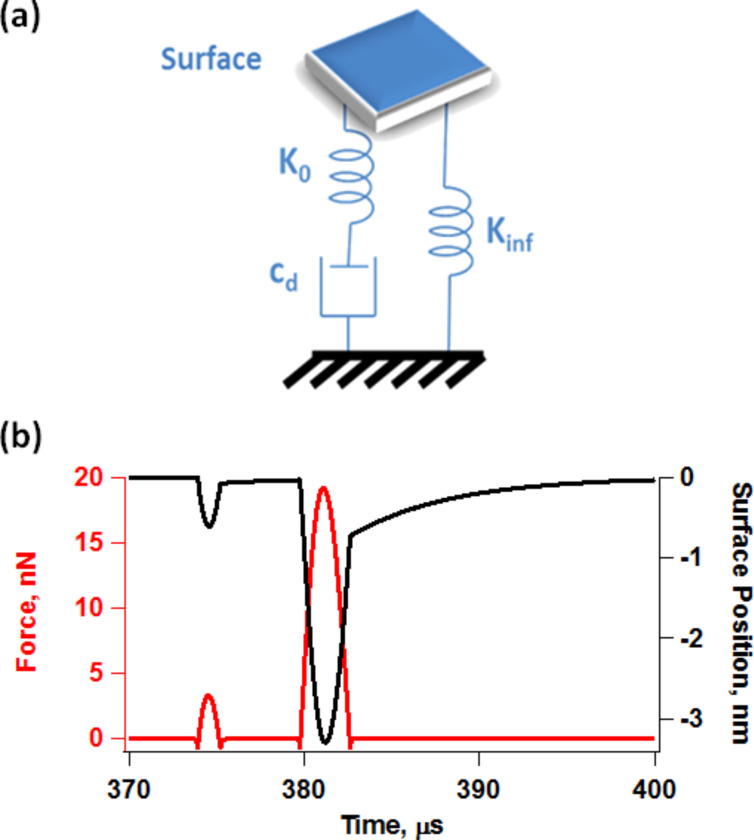
(a) Standard linear solid model; (b) illustration of tip–sample impact force trajectory and surface recovery for a bimodal imaging case.

The repulsive tip–sample forces were accounted in most simulations through a standard linear solid (SLS) model (Figure 11) [11,40], but Hertzian contacts [41] were also used in some cases. Long-range attractive interactions were included but for liquid environment simulations were assumed to be screened down to ≈10% of their typical value in air for a tip radius of curvature of 10 nm and a Hamaker constant of 2 × 10^−19^ J (no screening was considered for the simulations in air). Unless otherwise indicated, the trajectories shown indicate the true eigenmode or tip response, as opposed to the photodetector reading, which does not necessarily correspond to the true trajectory (as discussed in the text).
